# Experience with cardiac MR imaging of patients with legacy ICDs or pacemakers

**DOI:** 10.1186/1532-429X-15-S1-T4

**Published:** 2013-01-30

**Authors:** Kelley J Thounlasenh, Robert W Prost, Jason Rubenstein

**Affiliations:** 1 MRI, Froedtert Hospital, Milwaukee, WI, USA; 2 Radiology, Medical College of Wisconsin, Milwaukee, WI, USA

## Background

Previously, non-MR compatible pacemakers (PM) and cardiac defibrillators (ICDs) have been considered contraindication for MRI scanning. Device failure, lead tip heating, and patient discomfort have been of concern. Few institutions are currently scanning patients with these devices due to these concerns or lack of monitoring support. Additionally, cardiac imaging has been a unique challenge due to metal image artifact. This report will demonstrate techniques to improve diagnostic quality of cardiac imaging of a patient with pacemaker or ICD.

## Methods

The protocol for MR imaging of non-MR compatible devices includes an analysis of the risks versus benefits by the ordering physician and the EP Cardiologist monitoring the exam. Verifying type and patient dependence of the device should be known prior to scheduling exam. A chest x-ray is evaluated for pre-screening to identify abandon leads. Prior to entering the MRI suite, the device is interrogated by an EP Cardiologist and ECG and pulse oximetry monitoring is put on the patient.

**Figure 1:**
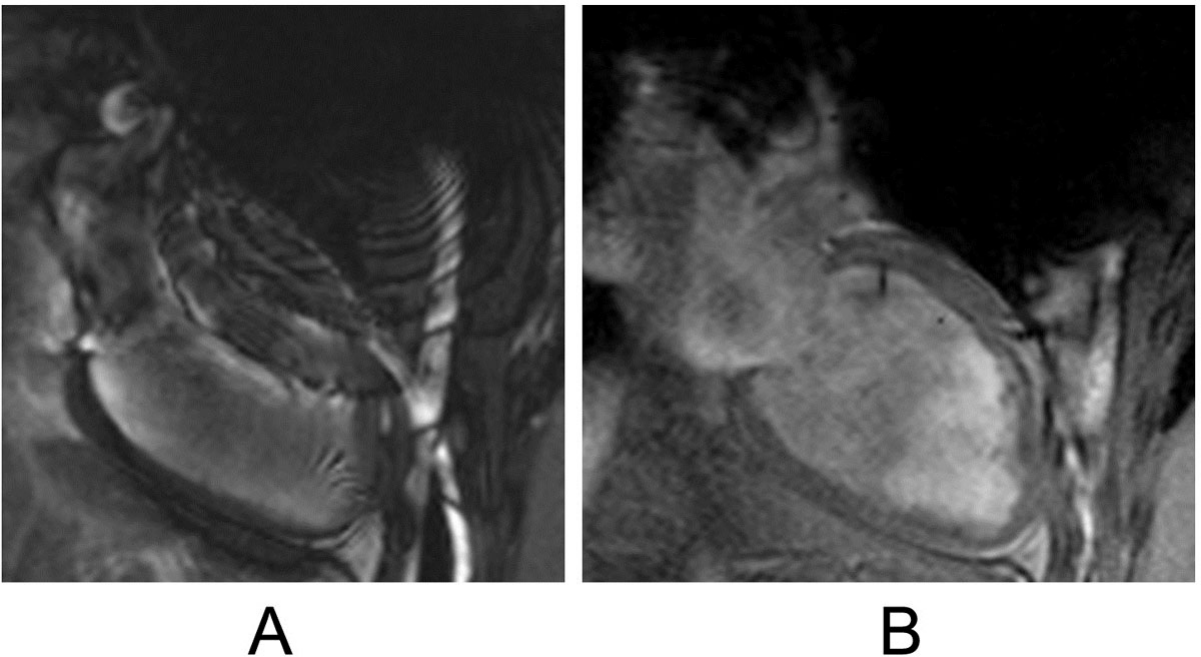
Single frames from 2-chamber cine sequences with similar field-of-view parameters, except panel A is steady-state free precession, and panel B is turbo gradient echo. Note metal artifact from the device generator obscuring the anterior wall in A.

**Figure 2:**
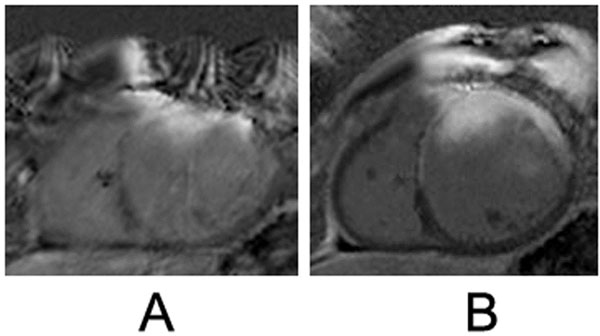
Short-axis delayed-enhancement images, with identical imaging parameters except for bandwidth (150 hertz/pixel in panel A and 606 in panel B). Note that anteroseptal delayed enhancement becomes evident only as anterior device generator metal artifact is reduced in panel B.

Cardiac imaging in patients with PM/ICDs is often limited by metallic artifact; more commonly from the pectoral generator than the intracardiac leads, presumably due to higher degree of ferrous material in the device generator. Steady-state free-precession imaging (SSFP) is subject to the most extreme artifacts; gadolinium delayed-enhancement images (Gre based) are more moderated affected. HASTE (fast single shot spin-echo) and perfusion (Gre based) pulse sequences are least affected by the device artifact. Our center has demonstrated that image quality can be improved by switching cine acquisitions from SSFP to Gradient Echo (Figure 1, A vs. B). Additionally, by moving from standard acquisition bandwidth to high bandwidth can reduce metal artifact in delayed-enhancement images (Figure 2, A vs. B). The generator can be pulled caudally and externally taped to reduce exposure to the cardiac imaging field. Also, breath-holds in deep inspiration can increase the heart-generator distance.

Most artifact occurs in the basal anterior segments; this should be taken into consideration if a patient has a known ischemic disease in these areas. Small patient size will increase the chance of generator artifact as well.

## Results

To date, 25 patients with non-MR compatible ICDs/PM have been scanned, including 10 cardiac studies. There have been no MR-related adverse outcomes. All scans have been at least partially diagnostic.

## Conclusions

Cardiac imaging of patients with pacemakers and ICDs can be safely and diagnostically completed to evaluate many disease processes with some adjustments to acquisition technique and parameters.

## Funding

N/A

